# Supplementary material for: Netrin signaling shapes sexually dimorphic neurite branching of a sex-shared interneuron in C. elegans
Source: bioRxiv. 2026 Oct 6:2026.09.29.755526. Preprint. [Version 1] doi: 10.64898/2026.09.29.755526 (PMC13691947; doi:10.64898/2026.09.29.755526)

bioRxiv preprint doi: https://doi.org/10.64898/2026.09.29.755526; this version posted October 6, 2026. The copyright holder for this preprint (which was not certified by peer review) is the author/funder, who has granted bioRxiv a license to display the preprint in perpetuity. It is made available under a CC-BY 4.0 International license.

Table S1. Transgenic strains used in this study

Strain name	Transgene name	DNA on array	Strain background	Source
EM1462	*bxIs29*	*eat-4p11::gfp + eat-4pA::wcherry + inx-18p::bfp*	*him-5(e1490)V*	Kim and Emmons (2017)
BHK186	*goyEx143; bxIs29*	*inx-18p::tra-2ic::sl2::mcherry (20ng/ul) + ttx-3p::gfp (50ng/ul) + DNA ladder(1kb plus) (30ng/ul)* – Line #1	*him-5(e1490)V*	This study
BHK191	*goyEx143; bxIs29*	*inx-18p::tra-2ic::sl2::mcherry (20ng/ul) + ttx-3p::gfp (50ng/ul) + DNA ladder(1kb plus) (30ng/ul)* – Line #2	*him-5(e1490)V*	This study
BHK185	*goyEx142; bxIs29*	*inx-18p::fem-3::sl2::mcherry (20ng/ul) + ttx-3p::GFP (50ng/ul) + DNA ladder(1kb plus) (30ng/ul)* – Line #1	*him-5(e1490)V*	This study
BHK188	*goyEx142; bxIs29*	*inx-18p::fem-3::sl2::mcherry (20ng/ul) + ttx-3p::GFP (50ng/ul) + DNA ladder(1kb plus) (30ng/ul)* – Line #2	*him-5(e1490)V*	This study
BHK151	*bxIs29*	*eat-4p11::gfp + eat-4pA::wcherry + inx-18p::bfp*	*him-5(e1490)V; unc-40(e271)I*	This study
BHK161	*bxIs29*	*eat-4p11::gfp + eat-4pA::wcherry + inx-18p::bfp*	*him-5(e1490)V; unc-6(ev400)X*	This study
BHK179	*bxIs29*	*eat-4p11::gfp + eat-4pA::wcherry + inx-18p::bfp*	*him-5(e1490)V; unc-5(e53) IV*	This study
BHK182	*goyEx138; bxIs29*	*inx-18p::unc-40cDNA (25ng/ul) + ttx-3p::GFP (25ng/ul) + DNA ladder (50ng/ul)*	*him-5(e1490)V; unc-40(e271)*	This study
BHK187	*goyEx144; bxIs29*	*inx-18p::ΔFNIII domain 4-5 (25ng/ul) + ttx-3p::GFP (25ng/ul) + DNA ladder(1kb plus) (50ng)* – Line #1	*him-5(e1490)V; unc-40(e271)*	This study
BHK190	*goyEx144; bxIs29*	*inx-18p::ΔFNIII domain 4-5 (25ng/ul) + ttx-3p::GFP (25ng/ul) + DNA ladder(1kb plus) (50ng)* – Line #2	*him-5(e1490)V; unc-40(e271)*	This study
BHK86	*otIs638*	*unc-6::SL2::NLS::YFP::H2B + ttx-3p::mcherry + pha-1(+)*	*him-5(e1490)V*	CGC
BHK198	*goyEx146; bxIs29*	*eat-4pA::tu813 (25ng/ul) + syg-1p::tu814 (25ng/ul) + ceh-22p::GFP (25ng/ul) + DNA ladder(1kb plus) (25ng/ul)* – Line #1	*him-5(e1490)V*	This study
BHK199	*goyEx146; bxIs29*	*eat-4pA::tu813 (25ng/ul) + syg-1p::tu814 (25ng/ul) + ceh-22p::GFP (25ng/ul) + DNA ladder(1kb plus) (25ng/ul)* – Line #2	*him-5(e1490)V*	This study
BHK201	*goyEx147; bxIs29*	*eat-4p11::tu813 (25ng/ul) + eat-4p11::tu814 (25ng/ul) + ceh-22p::GFP (25ng/ul) + DNA ladder(1kb plus) (25ng/ul)* – Line #1	*him-5(e1490)V*	This study
BHK203	*goyEx147; bxIs29*	*eat-4p11::tu813 (25ng/ul) + eat-4p11::tu814 (25ng/ul) + ceh-22p::GFP (25ng/ul) + DNA ladder(1kb plus) (25ng/ul)* – Line #2	*him-5(e1490)V*	This study
BHK208	*goyEx150; bxIs29*	*eat-4pA::tu813 (25ng/ul) + sax-3p::tu814 (25ng/ul) + ttx-3::GFP (25ng/ul) + DNA ladder(1kb plus) (25ng/ul)* – Line #1	*him-5(e1490)V*	This study
BHK209	*goyEx150; bxIs29*	*eat-4pA::tu813 (25ng/ul) + sax-3p::tu814 (25ng/ul) + ttx-3::GFP (25ng/ul) + DNA ladder(1kb plus) (25ng/ul)* – Line #2	*him-5(e1490)V*	This study

bioRxiv preprint doi: https://doi.org/10.64898/2026.09.29.755526; this version posted October 6, 2026. The copyright holder for this preprint (which was not certified by peer review) is the author/funder, who has granted bioRxiv a license to display the preprint in perpetuity. It is made available under a CC-BY 4.0 International license.

Table S2. The number of animals used in each experiment

Figures 1E and 1F	Wild-type 40 hours after hatching *n* = 13, 44 hours *n* = 20, 48 hours *n* = 18, 52 hours *n* = 22
Figure 2B	Wild-type *n* = 25, *Ex[inx-18p::tra-2ic]* #1 *n* = 18, *Ex[inx-18p::tra-2ic]* #2 *n* = 22
Figure 2C	Wild-type male *n* = 25, *Ex[inx-18p::tra-2ic]* #1 *n* = 18, *Ex[inx-18p::tra-2ic]* #2 *n* = 22, Wild-type hermaphrodite *n* = 20, *Ex[inx-18p::fem-3]* #1 *n* = 18, *Ex[inx-18p::fem-3]* #2 *n* = 25
Figures 3C and 3D	Wild-type *n* = 24, *unc-6(e400)* *n* = 25, *unc-40(e271)* *n* = 23, *unc-5(e53)* *n* = 25
Figures 4B and 4C	Wild-type *n* = 24, *unc-40(e271)* *n* = 23, *Ex[inx-18p::unc-40]* *n* = 24, *Ex[inx-18p::unc-40ΔFNIII4-5domain]* #1 *n* = 19, *Ex[inx-18p::unc-40ΔFNIII4-5domain]* #2 *n* = 22
Figure 5E	Wild-type *n* = 25, recCaspase (PCA) #1 *n* = 20, recCaspase (PCA) #2 *n* = 20, recCaspase (PHC) #1 *n* = 24, recCaspase (PHC) #2 *n* = 21, recCaspase (HOA) #1 *n* = 21, recCaspase (HOA) #2 *n* = 17
Figure 6A	Wild-type *n* = 20, *Ex[inx-18p::tra-2ic]* #1 *n* = 19, *Ex[inx-18p::tra-2 ic]* #2 *n* = 20
Figure 6B	Wild-type male *n* = 20, *Ex[inx-18p::tra-2ic]* male #1 *n* = 20, *Ex[inx-18p::tra-2ic]* male #2 *n* = 20, Wild-type hermaphrodite *n* = 20, *Ex[inx-18p::tra-2ic]* hermaphrodite #1 *n* = 20, *Ex[inx-18p::tra-2ic]* hermaphrodite #2 *n* = 20
Figure 6C	Wild-type male *n* = 33, *Ex[inx-18p::tra-2ic]* male #1 *n* = 35, *Ex[inx-18p::tra-2ic]* male #2 *n* = 35, Wild-type hermaphrodite *n* = 34, *Ex[inx-18p::tra-2ic]* hermaphrodite #1 *n* = 24, *Ex[inx-18p::tra-2ic]* hermaphrodite #2 *n* = 35
Figure S2B and S2C	Wild-type hermaphrodite L4 *n* = 25, Wild-type hermaphrodite Young adult *n* = 15

(which was not certified by peer review) is the author/funder, who has granted bioRxiv a license to display the preprint in perpetuity. It is m
available under a CC-BY 4.0 International license.

Figure S1

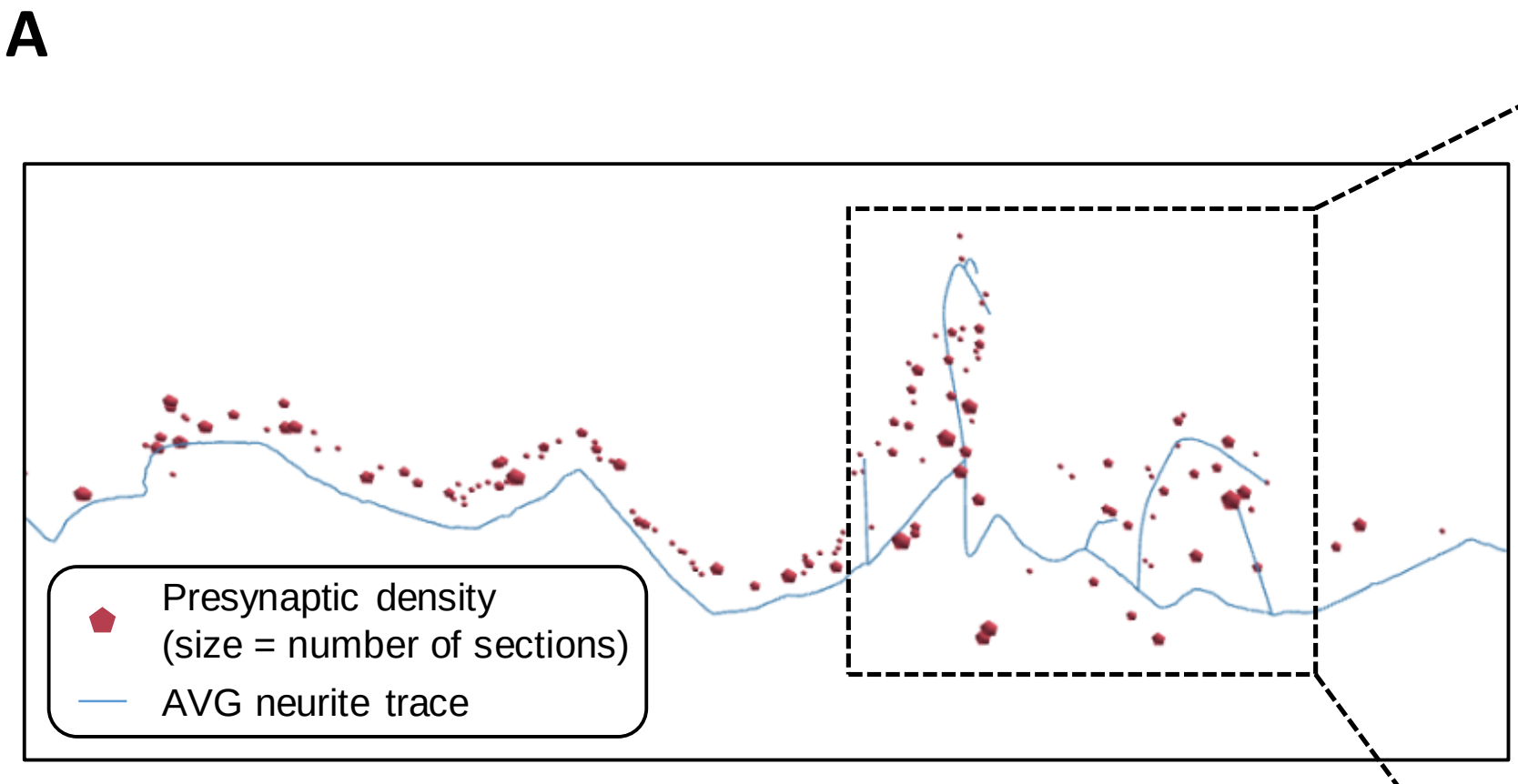

B Neurons presynaptic to AVG

Neuron	No. of sections	No. of synapses
PCA	30	17
PHC	28	7
HOA	27	19
PHB	14	8
R1B	14	5
R7B	6	3
PHA	4	2
R3B	3	1
PVV	3	1
PGA	2	1
AVH	1	1
PVX	1	1
CA07	1	1
R9A	1	1

(which was not certified by peer review) is the author/funder, who has granted bioRxiv a license to display the preprint in perpetuity. It is m
available under a CC-BY 4.0 International license.

Figure S2

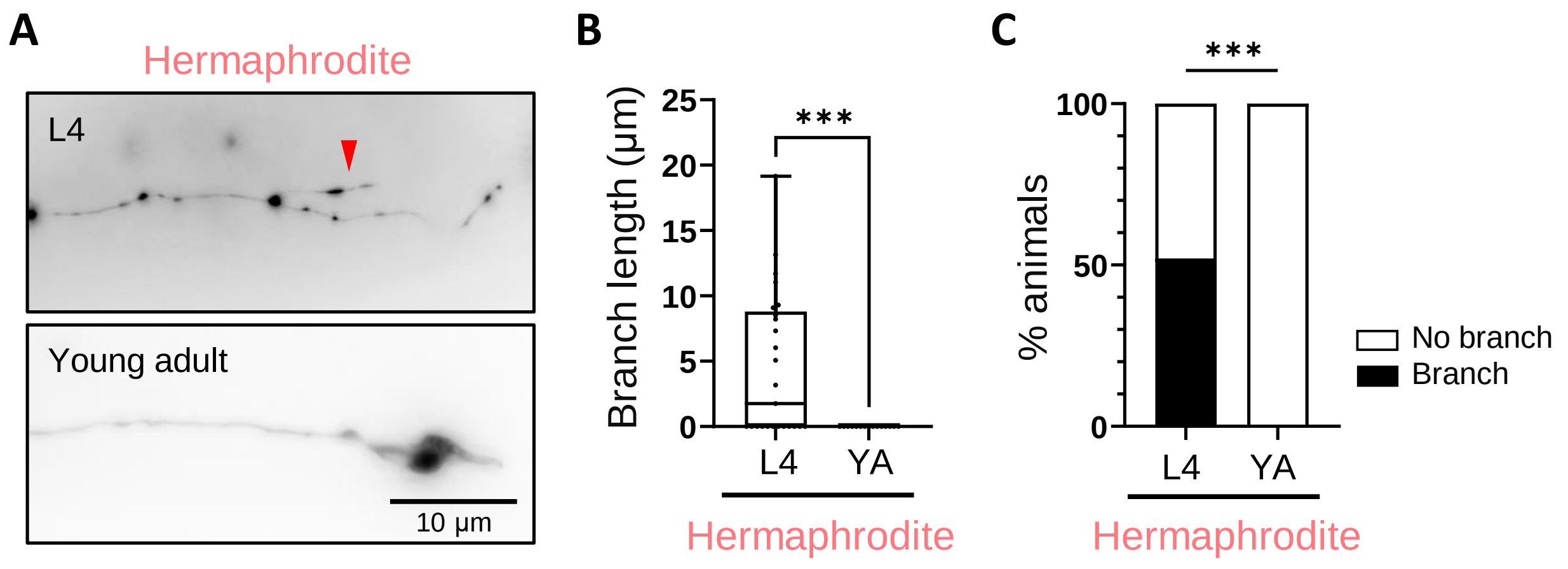

(which was not certified by peer review) is the author/funder, who has granted bioRxiv a license to display the preprint in perpetuity. It is m
available under a CC-BY 4.0 International license.

Figure S3

A

recCaspase

**PCA cell death**
eat-4pA + *syg-1p*
**HOA cell death**
eat-4pA + *sax-3p*
**PHC cell death**
eat-4p11 + *eat-4p11*

Promoter	Expression in the male tail
eat-4pA	**PCA, HOA**
syg-1p	R7-9A, **PCA**, PCB, PVX, DVC, PVT, PQR, PVR, DVA, PVQ, PVC, DA04-09, DD03-06, VA08-12, VD09-13, dBWML21-24, vBWML17-24, dgl1-8, adp, dsp, vsp
sax-3p	PHA, R2-5,7,9B, **HOA**, HOB, DVC, AVG, PVP, PVT, LUA, PVC, DB03-07, DD03-06
eat-4p11	**PHC**

B

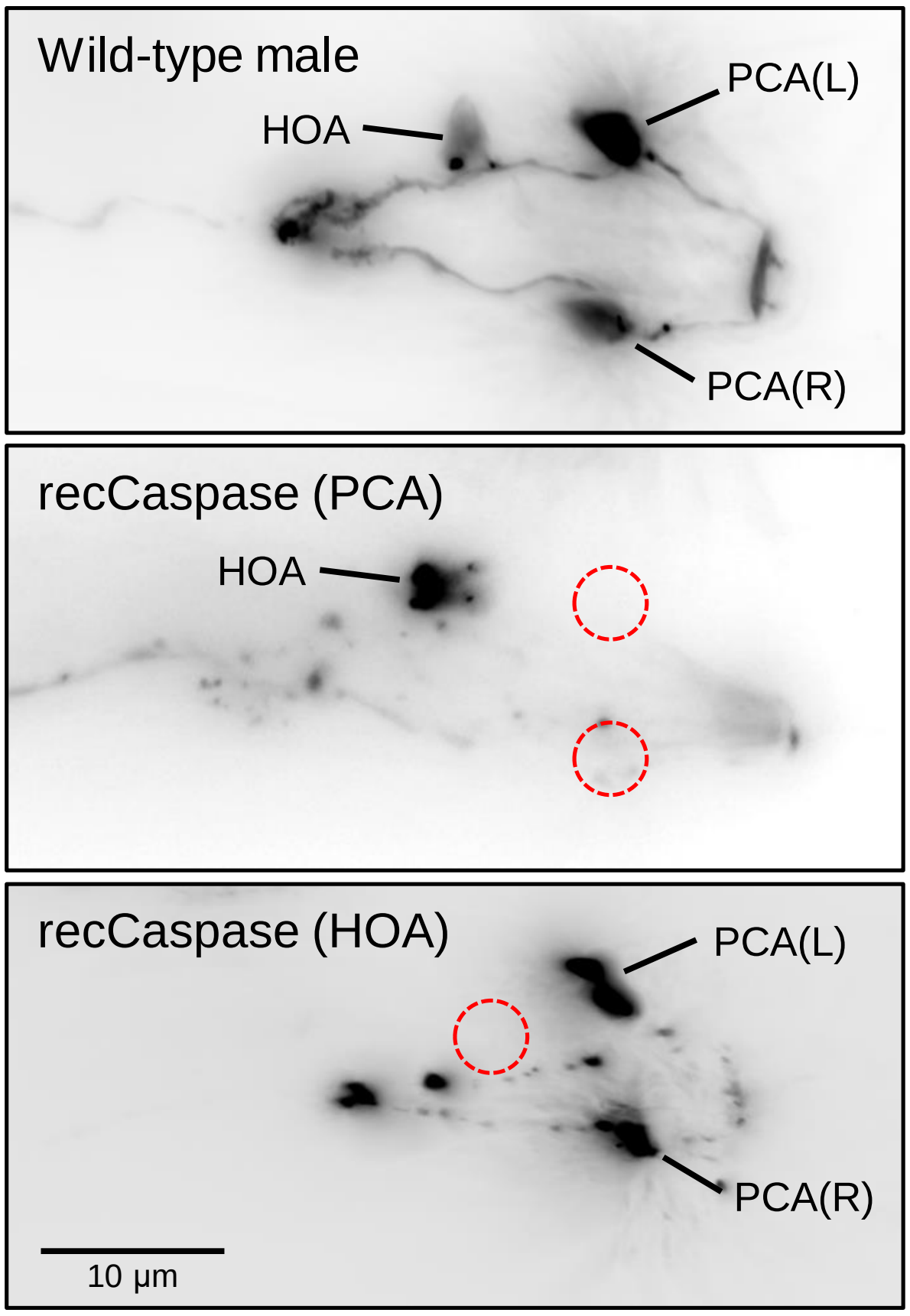

C

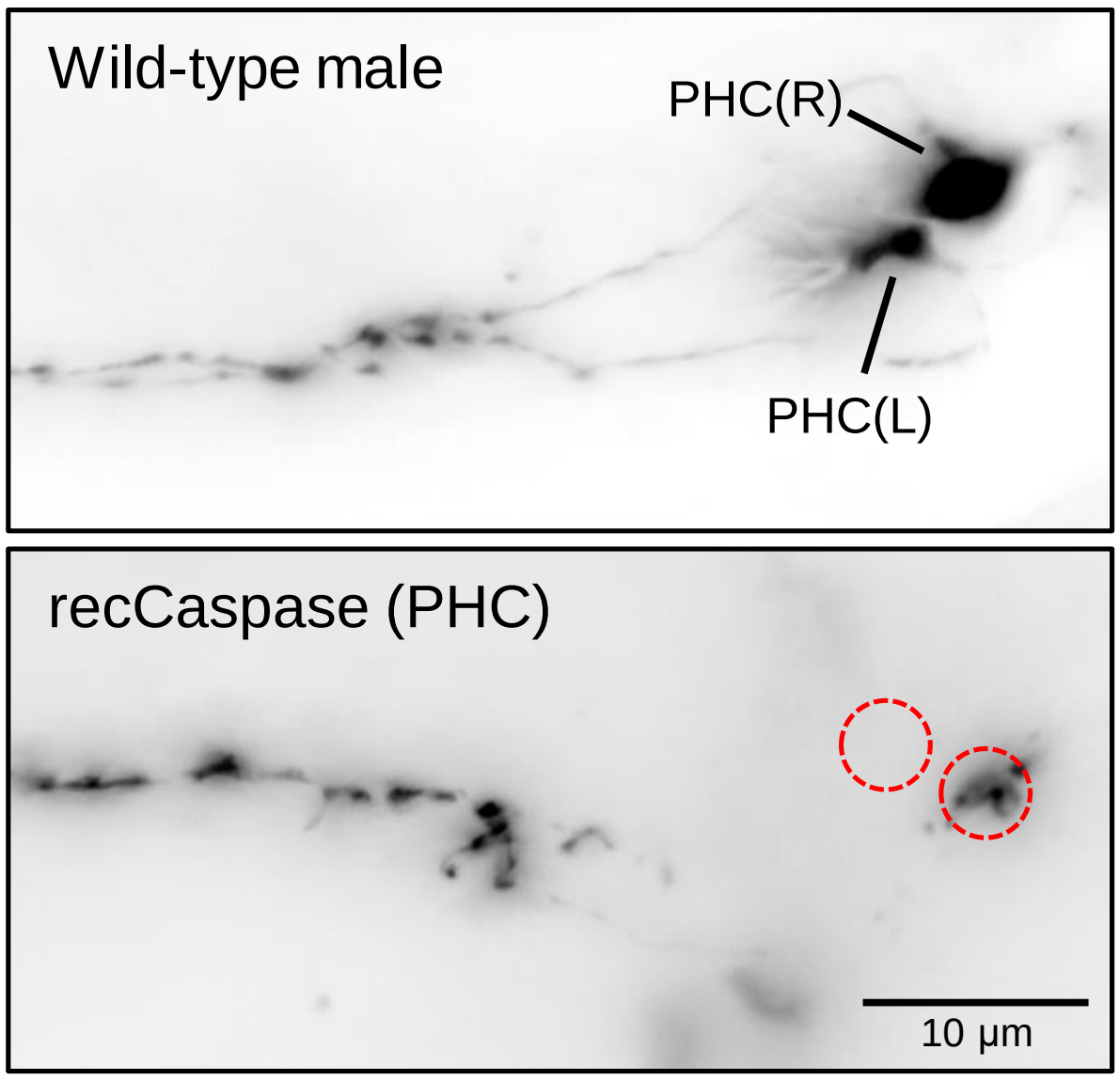

(which was not certified by peer review) is the author/funder, who has granted bioRxiv a license to display the preprint in perpetuity. It is m
available under a CC-BY 4.0 International license.

Figure S4

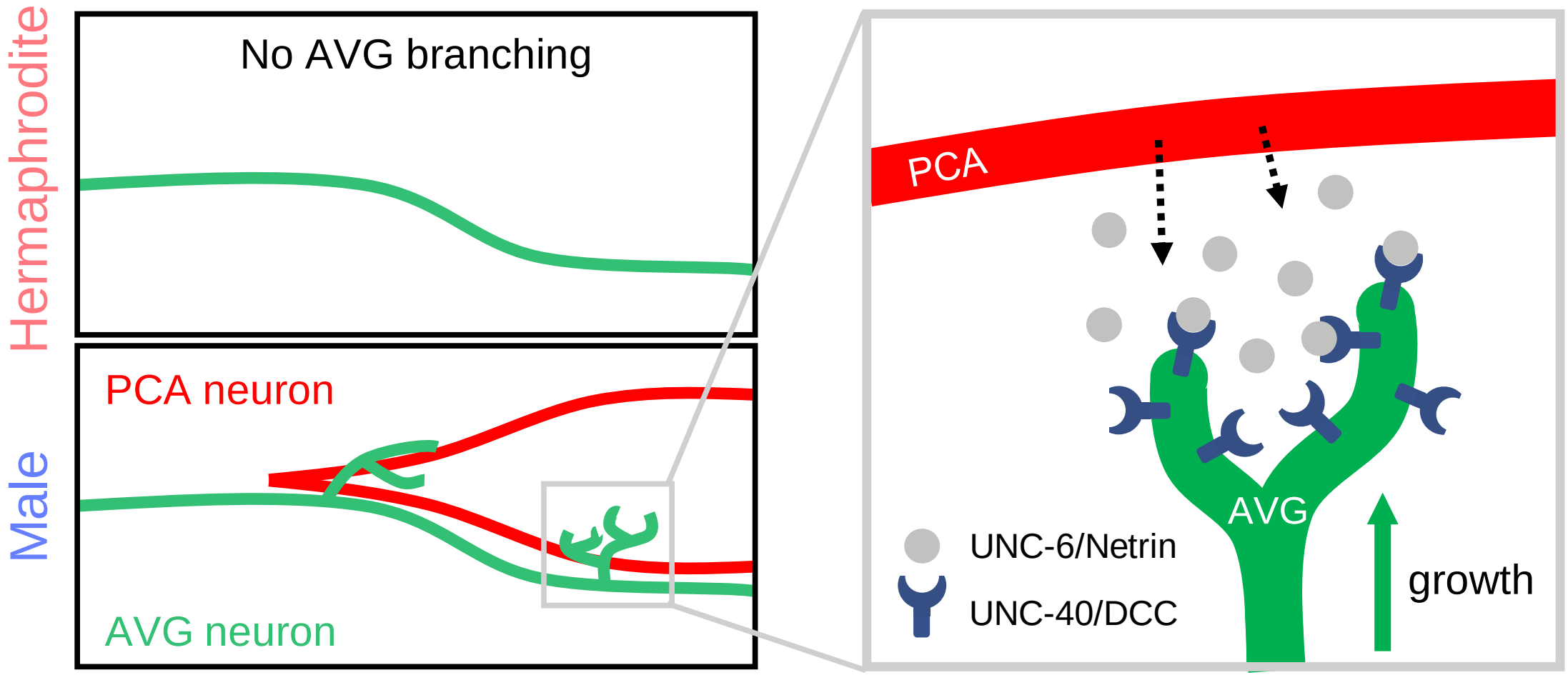

Supplement: Supplementary file 1 [file NIHPP2026.09.29.755526V1-supplement-1.pdf]
